# An assessment of the reporting of tapering methods in antidepressant discontinuation trials using the TIDieR checklist

**DOI:** 10.1007/s11096-023-01602-z

**Published:** 2023-06-03

**Authors:** Amy McGoldrick, Helen Byrne, Cathal Cadogan

**Affiliations:** School of Pharmacy and Pharmaceutical Sciences, Trinity College Dublin, Dublin 2, D02PN40 Ireland

**Keywords:** Antidepressants, Discontinuation, Reporting, Tapering

## Abstract

**Background:**

The importance of tapering is increasingly recognised when discontinuing antidepressant medication. However, no previous studies have examined the reporting of antidepressant tapering methods in published studies.

**Aim:**

The aim of this study was to assess the completeness of reporting of antidepressant tapering methods in a published systematic review using the Template for Intervention Description and Replication (TIDieR) checklist.

**Method:**

A secondary analysis was conducted of studies included in a Cochrane systematic review that examined the effectiveness of approaches for discontinuing long-term antidepressant use. The completeness of reporting of antidepressant tapering methods in included studies was independently assessed by two researchers using the 12 items from the TIDieR checklist.

**Results:**

Twenty-two studies were included in the analysis. None of the study reports described all checklists items. No study clearly reported what materials had been provided (item 3) or whether tailoring had occurred (item 9). With the exception of providing a name for the intervention or study procedures (item 1), only a minority of studies clearly reported on any of the remaining checklist items.

**Conclusion:**

The findings highlight a lack of detailed reporting of antidepressant tapering methods in published trials to date. This needs to be addressed as poor reporting could hinder replication and adaptation of existing interventions, as well as the potential for successful translation of effective tapering interventions into clinical practice.

## Impact statements

- Inadequate reporting of healthcare interventions in the scientific literature is a long-standing issue and hinders the potential for replication and implementation of evidence-based interventions in clinical practice.
- To date, no studies have been conducted into the completeness of reporting of antidepressant tapering methods in published trials.
- The study highlights a lack of detailed reporting of antidepressant tapering methods in published trials to date.
- These findings highlight the need for intervention reporting tools to be used by researchers in the write up of future studies to ensure tapering methods are adequately described, enabling their replication, adaptation and translation into practice.

## Introduction

Approximately 11% of the population take antidepressant medication daily, and the number of antidepressant prescriptions has almost doubled over the last decade [1, 2]. A key driving factor behind increased antidepressant prescribing rates is increased treatment duration [3]. There are growing concerns that antidepressants may be over-prescribed or prescribed for excessive durations. For example, it has been reported that 30–60% of long-term antidepressant prescriptions lack an evidence-based indication to support continued use and could potentially be deprescribed [4].

The importance of tapering is increasingly recognised when discontinuing antidepressant medication [5, 6]. This involves gradually reducing the dose over time to minimise the potential for withdrawal symptoms. However, guidance and instructions on tapering antidepressants varies [7]. In order for patients and healthcare professionals to be able to implement evidence-based tapering interventions, it is important that sufficient detail is reported in published study reports. However, a lack of detailed descriptions of healthcare interventions has been an issue in the scientific literature to date which hampers the potential for replication and can lead to research waste [8].

The Template for Intervention Description and Replication (TIDieR) checklist was developed to facilitate comprehensive reporting of interventions [9]. The checklist consists of 12 items considered essential to enable the replication of interventions (Table 1). The tool can also be used to evaluate the completeness of reporting in published studies included in systematic reviews [10, 11]. A recent Cochrane review examined the effectiveness and safety of approaches for discontinuing long-term antidepressant use [12]. However, the reporting of antidepressant tapering methods in included studies was not reviewed in detail.

**Table 1:** TIDieR checklist for assessment of intervention reporting

Item name	Item descriptor
1. Brief name	Provides a name or phrase that describes the intervention
2. Why	Describes the underlying rationale or theory underpinning the intervention
3. What (materials)	Describes any informational or physical or materials used in the intervention (including where the materials can be accessed)
4. What (procedures)	Describes the intervention’s procedures/activities and/or processes
5. Who provided	Provides information about the intervention providers (e.g. clinical expertise)
6. How (delivery mode)	Describes the intervention’s mode of delivery (including whether it was provided at an individual or group level)
7. Where (location)	Describes the locations(s) where the intervention was delivered
8. When and how much	Provides details on how often the intervention was delivered and over what time period
9. Tailoring	Describes whether any tailoring or adaption of the intervention occurred
10. Modifications	Describe any modification or changes to the intervention during the trial (what, why, when and how)
11. How well (planned)	Describes any strategies used to maintain or improve fidelity
12. How well (actual)	Reports on the extent to which the intervention was delivered as intended

### Aim

The aim of this study was to assess the completeness of reporting of tapering methods in antidepressant discontinuation trials in a published systematic review, using the TIDieR checklist.

### Ethics approval

The study involved application of the TIDieR checklist [9] to studies included in a systematic review and, therefore, ethical approval was not required.

## Method

A secondary analysis was conducted of studies identified in a Cochrane systematic review of randomised controlled trials (RCTs) comparing the effectiveness and safety of approaches for discontinuing long-term antidepressant use (> 6 months) versus continuation of the medication [12]. The review included 33 studies comprising interventions involving abrupt discontinuation (*n* = 13 studies), discontinuation involving tapering alone (*n* = 18 studies), discontinuation with minimal intervention (*n* = 1 studies), and discontinuation with psychological support (*n* = 4 studies).

For the current study, full-text articles of all studies included in the systematic review were obtained and independently reviewed by two researchers (AMcG, HB) to identify studies that implemented a tapering regimen as part of the discontinuation intervention. This was supplemented by a search for publications of studies that were classified as ‘ongoing’ in the original review and had since been completed. Using the TIDieR checklist (Table 1), the researchers independently extracted data from the intervention group in each of the identified studies to assess the completeness of reporting. In studies where the tapering intervention was supplemented by a co-intervention (e.g. psychological support), the TIDieR assessments focused specifically on the tapering component of the intervention. Each TIDieR checklist item was independently rated by the researchers as either ‘reported, ‘not reported’, or ‘unclear’ using the explanations provided for each item in the checklist. No modifications were made to the original checklist items. However, where studies made no reference to an assessment of adherence to tapering (item 11), item 12 was marked as ‘not applicable’. Study authors were not contacted for missing information as the focus of this assessment was the completeness of reporting in published study reports. Any disagreements between the researchers throughout the data extraction/assessment process were resolved through discussion and, consultation with a third researcher (CC) who assisted with reviewing and finalising all checklist assessments.

Cohen’s kappa (κ) co-efficient of inter-rater reliability was used to assess the level of agreement between the two independent assessors in applying the TIDieR checklist to each study prior to consensus discussion (< 0 = poor agreement; 0–0.20 = slight agreement; 0.21–0.40 = fair agreement; 0.41–0.60 = moderate agreement; 0.61–0.80 = substantial agreement; 0.81–1.00 = almost perfect agreement) [13]. The reported kappa co-efficient was calculated based on the degree of agreement between both assessors across all TIDieR checklist item assessments across included studies.

## Results

### Study characteristics

Twenty-two trials from the aforementioned Cochrane review implemented a tapering regimen as part of the discontinuation intervention and provided data for analysis [14–35], including one study that was originally classified as ‘ongoing’ and published subsequently [18].

Table 2 provides an overview of the characteristics of studies included in the current analysis. Briefly, these studies were published between 1982 and 2021. Study sample size ranged from 18 to 478 patients. Fourteen studies focused on a single antidepressant or class of antidepressants: serotonin and noradrenaline re-uptake inhibitors [SNRIs] (6 studies), selective serotonin re-uptake inhibitors [SSRIs] (1 study) and tricyclic antidepressants [TCAs] (7 studies). Sixteen studies involved the use of placebo substitution. Most studies (19 studies) reported taper durations ranging from 1 to 8 weeks. The most commonly reported taper duration was four weeks (10 studies). Eight studies provided details on tapering rates with reductions typically ranging from 25 to 50% at weekly or monthly intervals. Recurrence/relapse was the most commonly evaluated primary outcome (20 studies).

**Table 2:** Overview of study characteristics

Study ID	Country	Design	Setting	Participants	Sample size	Antidepressant	Interventions	Primary outcome
Bialos et al. [14]	US	RCT (double blind, two-armed)	Outpatient setting (mental hygiene clinic of a Veterans Administration Medical Centre)	Adult outpatients with a clinical diagnosis of depression	19	Amitriptyline	Intervention 1: Placebo (patients were tapered in three weekly decrements and then maintained on placebo) Intervention 2: Continuation of existing antidepressant treatment	Completion of the 6-month study protocol or recurrence of a depressive episode
Bockting et al. [15]	Netherlands	RCT (single blind, three-armed)	Community setting (recruitment via general practitioners, pharmacists, secondary mental health care, and media)	Adults with a history of major depressive disorder	289	SSRI (> 80%) Other reported antidepressants: SNRIs, tricyclic antidepressants, atypical antidepressants, monoamine oxidase inhibitors, > 1 antidepressant	Intervention 1: Antidepressant treatment and preventive cognitive therapy (PCT) Intervention 2: PCT with tapering of antidepressants (taper conducted over 4 weeks without placebo) Intervention 3: Continuation of existing antidepressant treatment	Time to recurrence
Cook et al. [16]	US	RCT (double blind, two-armed)	Outpatient setting (geriatric psychiatry clinic)	Adults with a history of major depressive disorder	18	Tricyclic antidepressant (amitriptyline, desipramine, doxepine, imipramine)	Intervention 1: Placebo (patients were gradually tapered off the medication over 4–8 weeks using placebo and then maintained on placebo) Intervention 2: Continuation of existing antidepressant treatment	Recurrence
DeRubeis et al. [17]	US	RCT (two-armed)	Outpatient setting (outpatient research clinics)	Adults with a history of major depressive disorder (chronic or recurrent)	292	Not reported	Intervention 1: Discontinuation of antidepressant treatment (without placebo over 4 weeks or longer if clinically necessary) and discontinuation of cognitive behavioural therapy (CBT) Intervention 2: Continuation of existing antidepressant treatment and discontinuation of CBT	Recurrence of major depressive episode
Duffy et al. [18]	UK	RCT (double blind, two-armed)	Primary care (general practices)	Adults with a history of depression who were taking antidepressants for ≥ 9 months	478	Citalopram (47%) Other reported antidepressants: sertraline, fluoxetine, mirtazapine	Intervention 1: Continuation of existing antidepressant treatment Intervention 2: Discontinuation of antidepressant treatment (50% dose reduction for first month followed by either half the dose of their usual medication or placebo, on alternate days and then placebo only from the third month onwards)	Time to recurrence
Eveleigh et al. [19]	Netherlands	cRCT (two-armed)	Primary care (family practice)	Adults with a history of long-term antidepressant use (> 9 months consecutively)	146	SSRI (> 70%) Other reported antidepressants: SNRI, TCA, or other non-TCA (excluding MAOI)	Intervention 1: Patient-specific letter sent to GP recommending antidepressant discontinuation (using gradual tapering programme) Intervention 2: Usual care	Successful discontinuation of long-term antidepressant use
Huijbers et al. [20]	Netherlands	RCT (two-armed)	Outpatient setting (secondary and tertiary psychiatric outpatient clinics)	Adults with a history of depressive disorder (full or partial remission)	249	SSRI (58%) Other reported antidepressants: SNRI, MAOI, mirtazapine	Intervention 1: Mindfulness-based cognitive therapy (MBCT) followed by discontinuation of existing antidepressant treatment (gradually over a recommended period of 5 weeks) Intervention 2: MBCT and continuation of existing antidepressant treatment	Relapse/recurrence
Keller et al. [21]	US	RCT (double blind, two-armed)	Outpatient setting (academic medical centres and clinical research centres)	Adults with a history of depressive disorder (chronic major depression or dysthymic disorder with a concurrent diagnosis of major depression)	161	Sertraline	Intervention 1: Continuation of sertraline treatment Intervention 2: Placebo (patients were tapered using placebo substitution at a rate of 50 mg/week and then maintained on placebo)	Time to recurrence of a major depressive episode
Keller et al.[22]	US	RCT (double blind, two-armed)	Outpatient setting (unspecified)	Adults with a history of major depressive disorder (chronic or recurrent)	131	Venlafaxine	Intervention 1: Continuation of venlafaxine treatment Intervention 2: Placebo (patients were tapered using placebo substitution over a 4-week period and then maintained on placebo)	Time to recurrence of major depressive disorder
Khan et al. [23]	US	RCT (double blind, three-armed)	Outpatient setting (clinical research centres)	Adults with a history of major depressive disorder (acute or recurrent)	361	Desvenlafaxine	Intervention 1: Continuation of desvenlafaxine treatment Intervention 2: Abrupt discontinuation of desvenlafaxine using placebo substitution Intervention 3: Tapering (patients were tapered using 25 mg desvenlafaxine daily for one week and then maintained on placebo)	Withdrawal symptoms
Kocsis et al. [24]	US	RCT (double blind, two-armed)	Outpatient setting (psychiatric outpatient clinic)	Adults with dysthymia with or without current major depression or major depression-chronic subtype	53	Desipramine	Intervention 1: Continuation of desipramine treatment Intervention 2: Placebo (patients were tapered by approximately 25% per week over four weeks and then maintained on placebo)	Relapse/Time to relapse
Kocsis et al. [25]	US	RCT (double blind, two-armed)	Outpatient setting (unspecified)	Adults with a history of major depressive disorder	336	Venlafaxine	Intervention 1: Continuation of venlafaxine treatment Intervention 2: Placebo (patients were tapered over four weeks and then maintained on placebo)	Recurrence
Kupfer et al. [26]	US	RCT (two-armed)	Not reported	Adults with a history of depression	20	Imipramine	Intervention 1: Continuation of imipramine treatment Intervention 2: Placebo (patients were tapered using a dosage reduction schedule involving a reduction of 33% per week for the first 3 weeks then maintained on placebo)	Time to recurrence
Kuyken et al. [27]	UK	RCT (two-armed)	Primary care	Adults with a history of depression	123	SSRI (58%) Other reported antidepressants: TCA or combination	Intervention 1: Continuation of existing antidepressant treatment Intervention 2: MBCT followed by support to taper (exact tapering regimen determined by patient and primary care physician)	Time to relapse/recurrence
Kuyken et al. [28]	UK	RCT (single blind, two-armed)	Primary care (general practices)	Adults with a history of recurrent major depressive disorder	424	Not reported	Intervention 1: Continuation of existing antidepressant treatment Intervention 2: MBCT followed by support to taper (exact tapering regimen determined by patient and primary care physician)	Time to relapse/recurrence
Mavissakalian et al. [29]	US	RCT (double blind, two-armed)	Outpatient setting (unspecified)	Adults with a history of panic disorder with agoraphobia	56	Imipramine	Intervention 1: Continuation of existing imipramine treatment Intervention 2: Placebo (patients were tapered by 25% each week over four weeks and then maintained on placebo)	Relapse
Mavissakalian et al. [30]	US	RCT (double blind, two-armed)	Outpatient setting (unspecified)	Adults with a history of panic disorder with agoraphobia	18	Imipramine	Intervention 1: Continuation of existing imipramine treatment Intervention 2: Placebo (patients were tapered by 25% each week over four weeks and then maintained on placebo)	Relapse
Montgomery et al. [31]	US and Europe	RCT (double blind, two-armed)	Outpatient setting (psychiatric centres)	Adults with a history of recurrent major depressive disorder	235	Venlafaxine	Intervention 1: Continuation of existing venlafaxine treatment Intervention 2: Placebo (patients were tapered over two weeks and then maintained on placebo)	Recurrence
Perahia et al. [32]	France, Germany, Italy, Russia, Sweden, US	RCT (double blind, two-armed)	Outpatient setting (study centres)	Adults with a history of recurrent major depressive disorder	288	Duloxetine	Intervention 1: Continuation of existing duloxetine treatment Intervention 2: Placebo (patients were tapered over four weeks and then maintained on placebo)	Time to recurrence
Rickels et al. [33]	US	RCT (double blind, two-armed)	Outpatient setting (central clinic in a university)	Adults with a diagnosis of generalised anxiety disorder	136	Venlafaxine	Intervention 1: Continuation of existing venlafaxine treatment Intervention 2: Placebo (patients were tapered by 75 mg weekly over 1–3 weeks with reduction to 37.5 mg during final week and then maintained on placebo)	Relapse
Segal et al. [34]	Canada	RCT (three-armed)	Outpatient setting (mental health clinics)	Adults with a diagnosis of major depressive disorder	84	Citalopram (or sertraline if not tolerated) Venlafaxine or mirtazapine if documented failure of SSRI	Intervention 1: Continuation of existing antidepressant treatment Intervention 2: Taper (patients were tapered over four weeks without placebo substitution) and MBCT Intervention 3: Placebo (patients were tapered over four weeks using placebo substitution and then maintained on placebo) and clinical management	Time to relapse/recurrence
Stewart et al. [35]	US	RCT (double blind, two-armed)	Outpatient setting (research clinic)	Adults with a diagnosis of depression (major depression, dysthymia, or both) and definite/probable atypical depression	60	Imipramine, phenelzine	Intervention 1: Continuation of existing antidepressant treatment Intervention 2: Placebo (patients were tapered over two weeks and then maintained on placebo)	Recurrence

### TIDieR reporting

**Fig. 1:**
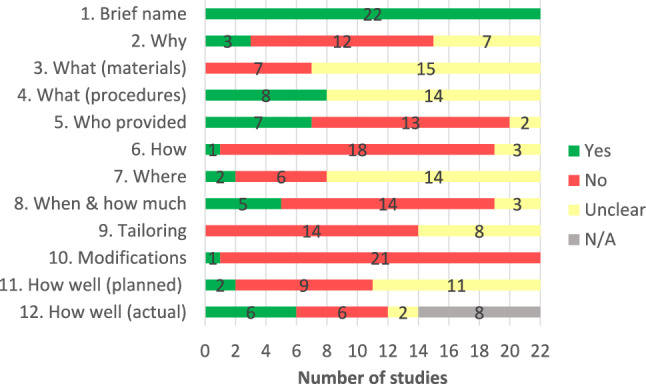
Summary overview of TIDieR checklist assessments

A summary of the finalised results from the TIDieR assessments is shown in Fig. 1, with a breakdown for each individual study provided in Table 3.

**Table 3:** TIDieR checklist assessments for individual studies (*n* = 22)

Study ID	TIDieR items (defined in Table 1)											
	1	2	3	4	5	6	7	8	9	10	11	12
Bialos et al. [14]	Yes	Unclear	Unclear	Unclear	No	No	No	No	No	No	Unclear	Unclear
Bockting et al. [15]	Yes	No	Unclear	Unclear	Yes	No	No	No	Unclear	Yes	Unclear	Yes
Cook et al. [16]	Yes	No	Unclear	Unclear	No	No	Unclear	No	No	No	No	N/A
DeRubeis et al. [17]	Yes	No	No	Unclear	No	No	Unclear	No	Unclear	No	No	N/A
Duffy et al. [18]	Yes	Yes	Unclear	Yes	Yes	Yes	Yes	Yes	No	No	Yes	Yes
Eveleigh et al. [19]	Yes	Unclear	Unclear	Unclear	Yes	Unclear	Unclear	No	Unclear	No	No	Yes
Huijbers et al. [20]	Yes	No	Unclear	Unclear	Yes	No	Unclear	Unclear	No	No	Unclear	Yes
Keller et al. [21]	Yes	No	No	Yes	No	No	Unclear	Unclear	No	No	No	N/A
Keller et al.[22]	Yes	No	No	Unclear	No	No	No	No	No	No	No	N/A
Khan et al. [23]	Yes	Unclear	Unclear	Yes	No	No	Unclear	No	No	No	No	N/A
Kocsis et al. [24]	Yes	Unclear	Unclear	Yes	No	No	Unclear	Yes	No	No	Yes	No
Kocsis et al. [25]	Yes	No	Unclear	Unclear	No	No	No	No	No	No	No	N/A
Kupfer et al. [26]	Yes	No	Unclear	Yes	No	No	No	No	Unclear	No	Unclear	Unclear
Kuyken et al. [27]	Yes	Unclear	Unclear	Unclear	Yes	No	Unclear	No	Unclear	No	Unclear	Yes
Kuyken et al. [28]	Yes	Unclear	Unclear	Unclear	Yes	No	Unclear	No	Unclear	No	Unclear	Yes
Mavissakalian et al. [29]	Yes	No	Unclear	Yes	No	Unclear	Unclear	Yes	No	No	Unclear	No
Mavissakalian et al. [30]	Yes	No	Unclear	Yes	No	Unclear	No	Yes	No	No	Unclear	No
Montgomery et al. [31]	Yes	Yes	No	Unclear	No	No	Unclear	No	No	No	No	N/A
Perahia et al. [32]	Yes	Yes	No	Unclear	No	No	Unclear	No	Unclear	No	No	N/A
Rickels et al. [33]	Yes	No	Unclear	Yes	Unclear	No	Yes	No	Unclear	No	Unclear	No
Segal et al. [34]	Yes	Unclear	No	Unclear	Yes	No	Unclear	Yes	No	No	Unclear	No
Stewart et al. [35]	Yes	No	No	Unclear	Unclear	No	Unclear	Unclear	No	No	Unclear	No

*N/A* = Not applicable

None of the study reports described all checklists items. No study clearly reported what materials had been provided (item 3) or whether tailoring had occurred (item 9). With the exception of providing a name for the intervention or study procedures (item 1), only a minority of studies clearly reported on any of the remaining checklist items.

Only three studies provided a clear rationale for tapering and, where applicable, the use of placebo as part of the tapering process (item 2). Apart from referring to the use of placebo medication, studies provided limited details on the use of any additional materials that were provided to patients (item 3). In cases where a tapering resource or information letter was referred to, no information was provided on where these materials could be accessed.

Eight studies clearly reported on the tapering regime (item 4) in terms of both the taper rate and duration. Seven studies reported details of the healthcare providers involved in delivering the intervention (item 5), which were typically general practitioners (GPs) and/or psychiatrists. Only one study clearly reported on how the intervention was delivered (item 6) whereby a hospital pharmacy posted the medication to either patients’ home or GP surgery at fixed intervals. Only two studies clearly reported on where the intervention was delivered (item 7). Five studies reported on how frequently patients were seen during the tapering process (item 8) which was typically monthly. Only one study reported details of a modification to the intervention (item 10), whereby the tapering process was conducted over six months as opposed to the recommended four-week timeframe as most patients did not find the latter to be feasible. Two studies described methods for assessing adherence to study procedures (item 11) and a further six studies reported on the number of participants that adhered to the tapering regime without detailing how adherence was assessed (item 12).

### Inter-rater reliability of TIDieR scoring

Cohen’s kappa was 0.452, indicating moderate level of agreement between both independent applications of the checklist [13]. On further review, it was found that most of the differences between the independent assessors occurred in the scoring of items as ‘unclear’ versus ‘no’ due to the lack of detail in study reports. Merging these assessments resulted in substantial agreement between the assessors (κ = 0.72).

## Discussion

### Statement of key findings

This study assessed the completeness of antidepressant tapering interventions in studies included in a Cochrane review [12] using the TIDieR checklist and found that the descriptions of tapering methods in published studies lacked detail. This could negatively impact the potential for replication in future studies and implementation of effective tapering interventions in clinical practice.

### Strengths and limitations

To our knowledge, this is the first study to assess the completeness of reporting of antidepressant tapering methods in randomised controlled trials. The TIDieR checklist was a useful tool for the assessment of intervention reporting as it highlighted specific aspects of antidepressant tapering methods which require more detailed descriptions in future studies. Each of the studies was assessed by two assessors working independently. Although the initial inter-rater reliability assessment was moderate, this was found to be largely due to the lack of detailed reporting in study reports and the associated challenge of categorising checklist assessments as ‘not reported’ or ‘unclear’. It must be noted that the scope of this analysis was limited to the reporting of tapering interventions in studies included in a single Cochrane review that focused on antidepressants and with the exception of searches for publications of studies that were classified as ‘ongoing’ in the original review, no updated searches were performed. It is possible that other intervention arms in the included studies may have been reported in greater detail. Finally, most of the included studies were published before the TIDieR checklist was made available.

### Interpretation

The subject of antidepressant tapering is receiving increasing attention [5]. However, the combined findings of both the original review and the current analysis highlight considerable deficits with the existing evidence base. As noted in the original review, based on the rather limited body of available evidence, no firm conclusions could be made about the effectiveness and safety of the approaches studied to date for discontinuing long-term antidepressant use [12]. The current analysis highlights the challenges in learning from and building on the existing evidence base regarding tapering approaches due to considerable deficits in the completeness of intervention reporting. Nevertheless, the findings provide some interesting insights into previously evaluated antidepressant tapering methods.

Guidance on antidepressant withdrawal symptoms and tapering methods has changed considerably over time. For example, antidepressant withdrawal symptoms were previously described as mild and self-limiting, typically lasting only 1–2 weeks [36, 37]. However, this is increasingly refuted, and it is now recognised that many patients experience symptoms for extended periods after discontinuing antidepressants ranging from weeks to months, and even longer in some cases [38, 39]. This highlights the importance of tailoring tapering approaches according to individual needs, which none of the included studies adequately reported on. The topic of appropriate tapering rates and durations is the subject of much discussion. Whereas previously it was thought that antidepressants could be tapered in fixed linear increments over 2–4 weeks, as was the case in many of the included studies, this is no longer considered the correct approach [3, 5]. Hyperbolic tapering regimens are now increasingly being recommended for antidepressant discontinuation in order to achieve linear reductions in pharmacological effect and mitigate against potential withdrawal symptoms [40, 41]. However, none of the included studies reported adopting such an approach and therefore further evaluations of hyperbolic tapering regimens are required.

The use of placebo substitution as part of the tapering intervention was common across included studies. However, only one study clearly reported a rationale for including a placebo which related directly to the study’s aims of evaluating the pharmacological effect of maintenance antidepressant treatment in preventing relapse of depression [18]. As most of these studies involved double blind conditions, the feasibility and acceptability of a tapering approach involving placebo substitution in real-world clinical practice is unclear. The use of placebos in clinical practice raises important ethical questions, particularly in terms of the potential for patient deception and loss of autonomy in the treatment decision process [42]. Open-label placebos (i.e. placebos without deception) have been proposed as an alternative to standard placebo approaches [43, 44]. However, very few studies have examined the use of open-label placebo medication in treating depression to date [45]. The role of open-label placebo as part of antidepressant tapering regimens also requires further investigation.

In addition to the issues outlined above with the reported tapering methods, very limited information was provided across included studies in terms of any additional tapering resources and supports that were provided to study patients or healthcare professionals. This is important to address as the lack of available practical evidence-based resources means that patients are increasingly turning to the internet and online discussion forums for tapering-related advice and support [46–48]. Healthcare professionals have also reported a lack of support and practical guidance as barriers to assisting patients with discontinuing long-term antidepressant use [49, 50]. The inclusion of intervention resources in published reports of studies targeting other psychotropic medication, such as benzodiazepines, has enabled replication and adaptation of existing interventions in other studies [51, 52].

Finally, examining and reporting on the intervention’s implementation, such as fidelity to the intervention/study procedures and whether any modifications occurred is also critical to providing insights into any issues with intervention adherence or deviations from study protocols. For example, in the only study to report on a modification to the tapering intervention, it was found that a four-week taper was not feasible for patients and that a longer taper duration was required [15]. These types of insights provide important learnings for future researchers. Future studies of tapering interventions may wish to consider the inclusion of a process evaluation which can provide detailed insights into an intervention’s implementation, as well other relevant factors such as the intervention’s mechanism of impact and the context within which it is delivered [53].

### Further research

Further research is needed into optimal antidepressant tapering methods and researchers should ensure that future interventions are adequately reported to enable replication. This should include all aspects of a tapering plan, including the practical methods of achieving increasingly smaller doses in real world settings (e.g. pill cutting, liquid formulations) particularly in cases where a hyperbolic tapering regime is being used. Future research should also consider the completeness of reporting of tapering interventions for other classes of psychotropic medication (e.g. antipsychotics, benzodiazepines).

## Conclusion

The findings of this analysis highlight the need for adequate reporting of antidepressant tapering methods in future intervention studies. The lack of detailed reporting of tapering methods in research to date could ultimately contribute to research waste and delay advancements in this area as poorly reported interventions hinder replication and the potential for successful translation of effective tapering interventions into clinical practice. Future studies on antidepressant discontinuation could benefit from the use of reporting tools to ensure that details of tapering interventions are adequately reported.
